# The Effectiveness of Environmental Taxes in Reducing CO_2_ Emissions in Passenger Vehicles: The Case of Mediterranean Countries

**DOI:** 10.3390/ijerph18105442

**Published:** 2021-05-19

**Authors:** Mónica Meireles, Margarita Robaina, Daniel Magueta

**Affiliations:** 1 Iscte—Instituto Universitário de Lisboa, Business Research Unit (bru_iscte), 1649-026 Lisbon, Portugal; monica.meireles@iscte-iul.pt; 2 DEGEIT-Department of Economics, Management, Industrial Engineering and Tourism, University of Aveiro, Campus Universitário de Santiago, 3810-193 Aveiro, Portugal; mrobaina@ua.pt; 3 GOVCOPP—Research Unit in Governance, Competitiveness and Public Policy, 3810-193 Aveiro, Portugal; 4 ESTGA—School of Technology and Management, University of Aveiro, 3750-127 Águeda, Portugal

**Keywords:** carbon tax, passenger transport, Mediterranean countries, car emissions

## Abstract

The transport sector is the biggest source of CO_2_ emissions in Europe. It is responsible for over a quarter of all greenhouse gas emissions. Passenger vehicles, alone, account for nearly 41% of these emissions, resulting in human health impacts. To meet the Paris climate commitments, cars and vans should be decarbonized until 2050. Such a transformation requires general changes, such as how the vehicles are owned, taxed, and driven. The European Federation for Transport and Environment revealed that Mediterranean countries tend to emit less per vehicle compared to the northern and central Europeans. Intriguingly, this does not necessarily correspond to motorization rates. In this article, we assess whether the observed reductions in CO_2_ emissions in the Mediterranean countries can be attributed to vehicle taxation on CO_2_ emissions. We apply panel data econometric techniques using data on annual registrations from 2008 to 2018 and model the demand for new-vehicle purchases and their responsiveness to changes in both CO_2_-based taxation and circulation tax. Our results show the determinants of new-vehicle demand and the change in the emissions rate in each country under the taxation currently adopted. We found that fiscal policies can have an important role in reducing the emission in the Mediterranean countries.

## 1. Introduction

The transport sector is the biggest source of carbon dioxide (CO_2_) emissions in the European Union (EU), contributing to 27% of its total CO_2_ emissions, with passenger vehicles alone representing 41% [1]. The EU transport sector is currently highly dependent on fossil fuel-derived products, such as petrol and diesel, of which 93% is imported, and whose combustion results in Greenhouse Gas (GHG) emissions. These emissions have been increasing since 1990 and continue rising. If these transport emissions are not controlled, national 2030 climate goals will not be reached. To meet the 2050 Paris climate commitments, vehicle emissions must be reduced by 94% from 2005 levels [1]. Such a radical transformation cannot be achieved through incremental improvements to existing vehicles only. It requires general changes, such as how the vehicles are owned, taxed, and driven. Amongst others, shifting fiscal policies in favor of lower carbon vehicles and incentivizing car sharing, together with the reform of vehicle taxation, congestion charging, road pricing, parking constraints, public transports, walking, and cycling, could help in reaching Paris goals.

The empirical literature suggests that emissions from cars tend to be proportional to the wealth of the country. According to the Transport & Energy report [1], although in the EU-26, the emissions per capita compared to the GDP per capita follow the results found in the literature, the Eastern and Mediterranean countries tend to emit less per vehicle compared to the northern and central ones. Intriguingly, this does not necessarily correspond to motorization rates, since countries such as Italy have high motorization rates but low per vehicle emissions, while Denmark and Ireland, in comparison, have low motorization rates but high emissions per vehicle.

To reduce CO_2_ emissions from passenger vehicles, many countries have implemented command and control instruments, such as standards for CO_2_ emissions or for the fuel economy. Recently, those standards became more stringent, and there has been a shift towards market-based instruments, such as taxes on vehicle purchase and ownership.

The strategy of the EU to reduce CO_2_ emissions from passenger vehicles has been grounded on three pillars, namely, a voluntary agreement between the EU and vehicle manufacturers to reduce average CO_2_ emissions, a CO_2_ labelling directive, and a proposal to harmonize fiscal instruments regarding passenger cars across member countries. Even though the first two were already implemented and enforced since 1999, the latter is revealing itself to be much more difficult to be achieved because each country still has sovereignty in implementing their own tax policies on passenger vehicles.

Similar to many other sectors, originally taxes on passenger cars were focused on their ability to raise state revenues. Currently, countries have shifted this focus to the environment by introducing a CO_2_ component at the moment of registration of a new acquisition and/or in the form of a circulation tax paid each year. Countries use this kind of taxes as an instrument to mitigate the negative externalities that arise by the CO_2_ emissions with the subsequent effects on public health associated with air pollution.

The literature demonstrates several studies advocating vehicle taxes over standards to reduce emissions from new vehicles because of their cost-effectiveness, simplicity, and greater incentives for new technology adoption. According to the standard economic theory, the cost of reducing CO_2_ emissions is minimized when marginal abatement costs are equalized across firms and sectors; that is, when the equimarginal principle prevails, which occurs when market-based instruments, such as taxes, are applied. However, compared to the wide literature on fuel economy standards, very little empirical evidence reveals the effectiveness of taxes at reducing CO_2_ emissions of new passenger vehicles in the Mediterranean countries. This paper intends to identify whether the observed CO_2_ emissions rates in the Mediterranean countries can be associated with vehicle taxation on CO_2_ emissions.

This study is structured as follows. This section provides a literature review on the impact and effectiveness of policy instruments in reducing CO_2_ vehicles emissions. Section 2 presents the data and methodology employed. Section 3 addresses the results and discusses some policy implications, and Section 4 highlights the main findings.

### Policy Instruments to Reduce CO_2_ Vehicles Emissions: An Overview

The literature provides evidence that fiscal policies deliver strong incentives for car fleet renewal and influence consumers’ behavior towards more fuel-efficient passenger cars, allowing decreasing environmental damages. Currently, the main instruments are the registration taxes or purchasing taxes, the circulation taxes or annual taxes, and the fuel taxes. The registration tax is an up-front cost that, if differentiated according to the CO_2_ emissions of the vehicle, may have a strong impact on the consumer’s decision to buy a low carbon vehicle. Indeed, if this tax is exempted or reduced for electric vehicles, these environmental friendly cars become more affordable. The circulation tax is an annual fee adjusted according to the characteristics of the vehicle, including its engine power, horsepower, cylinder capacity, fuel type, and CO_2_ emissions. The impact of the latter is lower than the registration taxes because, in their decisions, consumers usually devote much more attention to the higher initial purchase price than to lower annual or monthly payment charges [2]. In both situations, the level of tax paid should be high enough to encourage the use of more fuel-efficient cars. Regarding the fuel taxes, they restrict the energy consumption in the transport sector, incentivizing consumers to buy more efficient cars and to change driving patterns, as well. Some other factors may affect the demand for more efficient engines and vehicle sizes, such as regulations limiting CO_2_ emissions from new passenger cars sold; the economic situation, as with increasing income levels, consumers tend to buy more expensive cars; and both taxes and incentives regarding either the purchase or use of vehicles [3].

Other taxes on the transportation industry were also proven effective in achieving the reduction in CO_2_ emission. A study conducted in the United States (US), when analyzing the effect of gasoline tax rates, demonstrates that higher gasoline taxes result in more travelling by public transport or even cycling and walking, and less travelling by private cars. Indeed, higher gasoline taxes were found to be a crucial instrument of environmental policy, as they incentivize the use of greener transportation modes [4]. Higher fuel taxes also lead consumers to prefer cars with better fuel efficiency, increasing the demand for more efficient vehicles [5]. Sterner [6] shows that fuel taxes slowed down the fuel demand growth and the respective CO_2_ emissions. Although fuel price elasticity can be quite high in the long run, it may be quite inelastic in the short run. In a meta-analytic study, Labandeira et al. [7] show that the average elasticity for gasoline in the short run is −0.293 while in the long run is −0.773. Additionally, in their own estimations, they found that in the short run, the elasticity for gasoline is always more inelastic than in the long run, even though the long run can present inelastic behavior. Therefore, if the European Union countries had not traditionally applied a high fuel taxation policy, contrasting with the U.S. low taxes policy, then the global fuel demand would have been larger, with all the GHG consequences.

A study in Japan found evidence that vehicle choices are quite inelastic to taxes not only on cars but also on fuel or distance. Nevertheless, emissions were shown to be more affected by taxes on gasoline than on vehicles. However, since taxes on purchasing cars have lower costs on consumers, the marginal abatement cost will be lower. Therefore, the analysis suggests that the marginal abatement costs from the use of distance-reducing taxes are higher as compared to the marginal abatement costs from induced changes in the consumers’ choices of vehicles. Thus, the most cost-effectiveness option is to tax each vehicle in proportion to its emission rate [8].

Bernard and Kichian [9], regarding the emissions in the transport sector in British Columbia, and measuring the impact of the tax on diesel users over 2008–2016, found that carbon taxes work in an effective way in reducing GHG emissions.

Watabe et al. [10], in turn, demonstrated that a rise in taxes on carbon gases, together with investment policies in infrastructure, could offer noticeable decreases in the emissions released. The authors analyze how the evolution towards lower emission vehicles in Japan (e.g., battery electric vehicles, natural gas vehicles, etc.) can contribute to the reduction of greenhouse gas (GHG) emissions. Their results show that, to achieve a considerable decrease in emissions, it is crucial to improve the infrastructure and to implement a high tax burden on CO_2_, since they strengthen the introduction of lower carbon vehicles.

Regarding the taxes on the vehicle registration and on the annual circulation focused on CO_2_ emissions, the literature also provides some studies with interesting results. In 2008, Finland was a pioneer in introducing this fiscal innovation by implementing a CO_2_ tax at the moment of the car purchasing. Since then, the average CO_2_ emissions from new passenger car registrations decreased considerably [11]. Knittel [12] points out that for the U.S., some other instruments, such as CAFE (Corporate Average Fuel Economy) standards, may play a useful role as a second-best policy. Nevertheless, he reinforces that setting a price on the externalities, namely through a carbon tax or cap-and-trade policy, would be desirable in addressing petroleum fuels externalities.

Portugal, following the example of other countries, also legislated on the introduction of a tax based on CO_2_ emissions when registering new vehicles. This tax strongly penalizes the most polluting vehicles, while presenting lower levels for diesel vehicles. As for the annual circulation tax, it is based on engine capacity, power, and number of cylinders, and has also a component related to CO_2_ emissions. As part of the government’s intervention in this area, there are special taxes on fuel consumption, as well as incentives for the purchase of electric vehicles through subsidies, exemption in some types of taxes, and other benefits such as special parking areas in cities [13]. In Spain, there is a total subsidy of €2000 for the replacement of an old car by a new more efficient one [14]. Valles-Gimenez and Zárate-Marco [15], using panel data for the different Spanish autonomous regions, investigated the effectiveness of environmental taxes. The authors show that this type of tax policy can indeed contribute to decreased CO_2_ emissions, although not in the desirable amount, suggesting that the country needs stronger measures if it wants to meet the targets set by the EU.

France employed a feebate system. Vehicles emitting less than 130 g CO_2_/km obtained a subsidy, while those emitting more than 160 g CO_2_/km had to pay a tax. The value of the subsidy varied amongst 200€ and 1000€, while the tax varied between 200€ and 2600€ [16]. To provide continued incentives for emission reductions, the thresholds declined in the years following the introduction of the feebate [17]. Currently, new passenger vehicles emitting less than 120 g CO_2_/km receive a subsidy at the moment of the car acquisition, whereas those emitting more than 138 g CO_2_/km pay a tax. The subsidy goes to a maximum of 6000€, while the tax ranges between 50€ and 20,000€ [18].

Although there are global trends in CO_2_ emissions for new passenger cars across all of the EU, there are significant differences within each member country. In the literature, some studies focus on the impact of vehicle taxes EU-wide, as for instance [3,19,20,21], and on national case studies, as for example [22,23,24,25].

In fact, especially in the passenger vehicle segment, the adoption of a tax to the price of new cars acquisition, combined with incentive policies to scrap older and therefore more polluting ones, has proven to be effective in promoting the demand for vehicles with lower CO_2_ emissions. Nevertheless, other types of measures can also contribute to achieve this goal. Denmark, with an integrated energy and transportation system, and with policies focused on the environment, attempts to achieve the desired decrease in emissions. Evaluating the level of effectiveness of these policies, Venturini et al. [22] conclude that taxes on CO_2_ emissions and on fossil fuels are the most efficient way of reducing carbon emissions in the transport sector, whereas the program of Mobility-as-a-Service is the most cost-effective of all those studied. For the Netherlands, Kok [23] makes a six-year assessment after the implementation of a CO_2_-based purchase tax and CO_2_-based tax incentives, between 2008 and 2013, for road and company taxes. His findings conclude that these fiscal policies resulted in an 11% reduction in CO_2_ emissions in 2013, shifting the country from the 12th position before the tax changes in 2007 to the first in Europe with the lowest CO_2_ emissions of new vehicles registered, and to the country with the highest share of electric vehicles in 2013. Without Dutch tax incentives based on CO_2_, the average emissions of new passenger vehicles would have ranked seventh instead. CO_2_-based tax incentives for company cars seem to have contributed the most to this result. Comparing tax incentives with the exogenous factors associated with the economic recession in 2008–2009, the tax incentives accounted for about two-thirds of the reduction in the average CO_2_ emissions, while the exogenous factors contributed to nearly one-third. In a study on France, Germany and Sweden, Klier and Linn [24] compare the consequences of vehicles taxes on registrations and CO_2_ emissions, and estimate the effect of these emissions’ reduction on the manufacturers’ profits. Their results show that taxes had a significant negative short-run impact on the registrations of new vehicles in all countries, even though the impact has been stronger in France, where it implemented CO_2_-based purchase taxes and subsidies, than in Germany and Sweden, where they imposed annual circulation taxes that increased linearly with the CO_2_ emissions. Regarding the manufacturers’ long-run reaction to these taxes, the authors did not find a strong evidence that the taxes influenced the CO_2_ emissions of the vehicles.

In a broader context, Dineen et al. [19] examined the case of EU member countries that base their vehicle’s fiscal policy on differentiated taxes according to the CO_2_ emissions, to understand better its effects in the emissions reduction. Their findings show that in most member states, there was an important decrease in CO_2_ emissions from new passenger cars since 2007 compared to the previous six years. This result suggests that EU regulations on vehicles, concomitant with the economic recession that occurred in 2008, affected consumer behavior. In general, the countries that adopted this type of taxation were the ones that most likely achieved higher decreases in CO_2_ emissions. Using a dynamic general equilibrium model and separating diesel from gasoline vehicles for 13 EU countries, Marrero et al. [20] points out that the dieselization did not help reduce fuel use or CO_2_ emissions of passenger vehicles due to the rebound effect. Indeed, since the diesel car is more efficient, it induces a more intensive use, generating negative impacts on CO_2_ emissions. Therefore, the authors suggest that a carbon-based tax discrimination of each fuel (lower for gasoline than for diesel) would be more successful in reducing CO_2_ emissions than a fuel efficiency-based tax or a purchase tax of new vehicles. Additionally, supported by previous studies, they argue that diesel generally presents greater external costs than gasoline, and thus Pigou taxes on the use of vehicles (based on the polluter pays principle), via fuel use or kilometers driven, should reflect it.

Countries with an important car manufacturing industry usually tend to impose lower registration taxes than countries that import passenger cars, as in Germany, France, UK, and Sweden. In contrast, countries such as The Netherlands, Denmark, Ireland, and Portugal have greater CO_2_-based acquisition taxes or other stricter low carbon tax incentives. The automotive industry in the European countries has a significant weight. Germany, Czech Republic, Hungary, and Slovakia are the most representative countries. Romania and Slovenia, for instance, are close to the European average, but they are expected to follow the same path as their neighbors due to additional local investments such as Ford in Romania and the launching of new plants. Similarly, Renault has a huge influence in Romania, while Seat (Volkswagen Group) does in Spain [1].

From the literature review, it is clear that there are several studies regarding the advantages of both vehicle and fuel taxation on the adoption of more environmentally friendly vehicles aimed at reducing CO_2_ emissions. However, to the best of our knowledge, few studies were implemented to analyze the effectiveness of passenger cars taxes based on CO_2_ in decreasing these emissions, and none were performed for the Mediterranean European countries. Another innovation is that this study used the CO_2_ emissions from transport activities, and the transport taxes specifically concerning the households, to understand better the private consumer behavior regarding the demand for new cars, and the CO_2_ emissions control. Therefore, this paper intends to identify whether the observed CO_2_ emissions rates in the Mediterranean countries can be attributed to CO_2_-based vehicle taxation. Table 1 presents a summary of the main vehicle taxes in the Mediterranean countries, based on data from [18].

## 2. Materials and Methods

### 2.1. Data

The present study uses annual data for the period between 2008 and 2018, and for the following Mediterranean European countries: Bulgaria, Croatia, Cyprus, Greece, Italy, France, Spain, Portugal, Slovenia, Malta, and Romania (according to [26] and to the available data). The period length was limited to the data availability. All data were collected from the Eurostat database.

It was intended to estimate two models: the first with the dependent variable Carbon Emissions from Transport activities by households (CO_2_) in tons and the second with the dependent variable New registrations of passenger cars in number of vehicles (NEW). This last variable is also considered as independent in the first model, and is expected to affect positively CO_2_ Emissions from Transport activities [24].

The following explanatory variables were considered:

- Gross Domestic Product (GDP), in million Euros at 2010 constant prices, was used as indicators of economic activity and is expected to affect positively both dependent variables [27];
- Car registration tax (REGTAX), in million Euros, is a tax that is paid only once, as it affects the first registration of the vehicle. In general, the registration tax has a substantial weight on the vehicle CO_2_ emissions, even though diesel vehicles taxes also have a list price component and a non-CO_2_ emissions component. A negative relationship is expected between this variable and the dependent variables [27];
- Transport taxes paid by households (TRTAX), in million Euros, include taxes related to the ownership and use of motor vehicles. This variable incorporates taxes on other transport equipment (e.g., planes, ships, or railway stocks) and related transport services (e.g., duties on charter or scheduled flights), as well as taxes on means of transport that are comparatively more environmentally friendly, for example railway rolling stock and public transport in general, as well as taxes on electric vehicles. Taxes on car insurance are also included, as they are taxes specific to vehicles and not general insurance taxes. Taxes on gasoline, diesel, and other transport fuels are included beneath energy taxes. Transport taxes also comprise the congestion charges or city tolls (levies that some cities impose to allow access to the city center) in case they are considered as a national accounts tax. It is expected that this variable has a negative impact on both CO_2_ and NEW [24].

The models proposed are the following linear regressions:

(1) $$\mathrm{Model}\;1:\;CO_{2it}=\alpha_{it}+\beta_{1}NEW_{it}+\beta_{2}GDP_{it}+\beta_{3}REGTAX_{it}+\beta_{4}TRTAX_{it}+\varepsilon_{it}$$

(2) $$\mathrm{Model}\;2:\;NEW_{it}=\alpha_{it}+\beta_{5}GDP_{it}+\beta_{6}REGTAX_{it}+\beta_{7}TRTAX_{it}+v_{it}$$

where subscripts *i* and *t* refer to country and time, respectively; $\beta_{1},\;\beta_{2},\beta_{3},\beta_{4},\;\beta_{5},\;\beta_{6},\beta_{7}$ are the slope parameters to be estimated; *Ɛ* and v are the error terms.

### 2.2. Methodology

The methodology selected is common in the literature to estimate panel data. In this case, apart from descriptive statistics and the Correlation Matrix, the following procedures were performed, after transforming variables data in logs (to reduce or remove the skewness of our original data, so that the statistical analysis results from this data become more valid): (i) Panel Unit Root tests, (ii) Panel Cointegration tests, (iii) Panel Fully Modified Least Squares (FMOLS) Model and Dynamic Ordinary Least Squares Estimator (DOLS), and (iv) Auto Regression Distributed Log Model (ARDL).

#### 2.2.1. Panel Unit Root Tests

Panel-based unit root tests are stronger than unit root tests based on individual time series, as suggested by recent literature. Consequently, in this study, the following Autoregressive AR (1) process for panel data was considered [28]:

(3) $$y_{it}=\rho_{i}y_{it-1}+\Delta_{i}X_{it}+\omega_{it}\;$$

where *i* = 1, 2, …, N represent countries observed over periods, *t* = 1, 2,…, T. $X_{it}$ are exogenous variables in the model including any fixed effects or individual trends, and $\rho_{i}\;$is the autoregressive coefficient. If $\rho_{i}\;$< 1, $y_{i\;}$ is said to be a weakly trend stationarily. Contrarily, if $\rho_{i}$ = 1, then $y_{i}$ comprises a unit root. $\omega_{it}$ is the stationary error term.

We performed four panel unit root tests: Levin, Lin, and Chu (LLC) [29]; Im, Pesaran, and Shin (IPS) [30]; and Fisher-type tests using Augmented Dickey-Fuller (ADF) and Phillips-Perron (PP) tests ([31,32]).

In order to test the null hypothesis, that all individual series of the panel hold a unit root, [29] proposed a panel-based ADF test where parameters are restricted, keeping them identical across sectional regions, as depicted in Equation (4):

(4) $$\Delta y_{it}=c_{i}+\rho_{i}y_{it-1}+\overset{k}{\underset{j=1}{\sum }}\rho_{j}y_{it-j}+\gamma_{it}$$

where *t* = 1, 2,…, T are the time periods, and *i* =1, 2,…, N the members of the panel. The LLC adopts the null hypothesis of $\rho_{i}=\rho \;$= 0 for all *i*, against the alternative $\rho_{i}=\rho_{2}=\ldots =\rho <0$ for all *i*, with the test based on the statistics $t_{\rho }=\frac{\hat{\rho }}{s.e.\left(\hat{\rho }\right)}$. However, one drawback is that ρ is constrained to be identical across regions under both the null and alternative hypotheses. Alternatively, ρ can be allowed to vary freely across cross-sections. $\gamma_{it}$ is the stationary error term. The IPS and Fisher-ADF and Fisher-PP tests are of this form.

Ref. [29] specifies a separate ADF regression for each cross section:

(5) $$\Delta y_{it}=\alpha y_{it-1}+\overset{p_{i}}{\underset{j=1}{\sum }}\beta_{ij}\;\Delta y_{it-1}+X_{it}^{\prime }\delta +\eta_{it}$$

In this test, H_0_: α_i_ = 0 is the null hypothesis, while the alternative hypothesis is expressed by: $H_{1}:\{\begin{array}{c} \alpha_{i}=0\;for\;i=1,2,\ldots ,N_{1}\\ \alpha_{i}<0\;for\;i=N+1,N+2,\ldots ,N \end{array}\;$which may be interpreted as a stationary non-zero fraction of the individual processes, with $\eta_{it}$ representing the stationary error terms.

References [31,32] proposed a different method to panel unit root tests results, deriving tests that combine the *p*-values from individual unit root tests [33]. If π*_i_* is defined as the *p*-value from any individual unit root test for cross-section *i*, then under the null of unit root for all *N* cross-sections, the asymptotic result holds as it follows:

(6) $$-2\overset{N}{\underset{i=1}{\sum }}\log\left(\pi_{i}\right)\to x_{2N}^{2}$$

Additionally, [8] proved that:

(7) $$Z=\frac{1}{\sqrt{N}}\overset{N}{\underset{i=1}{\sum }}\Phi^{-1}\left(\pi_{i}\right)\to N\left(0,1\right)$$

where $\Phi^{-1}\;$is the inverse of the standard normal cumulative distribution function.

The asymptotic *x^2^* and the standard normal statistics using ADF and PP individual unit root tests were used. Both the null and alternative hypotheses remain the same as for the IPS test.

#### 2.2.2. Panel Cointegration Tests

Once assured of the non-stationarity, the cointegration hypothesis of the series must be tested, which is generally transformed using the method proposed in [34]. This approach examines the residuals of a regression and asserts that there is cointegration if u_t_ ~ I(0). The pioneer contribution for this methodology was presented, among others, by [35,36,37,38], given the following equation:

(8) $$y_{it}=\alpha_{i}+\delta_{i}+\beta_{1i}x_{1i,t}+\beta_{2i}x_{2i,t}+\ldots +\beta_{ki}x_{ki,t}+\zeta_{it}$$

where *t* = 1, 2,…, *T* and *k* = 1, 2,…, *K*; the parameter α denotes the individual characteristics; *k* is the number of explanatory variables; and δ is the trend. It is further assumed that variables *y* and *x* are integrated of order one, that is, I(1). Thus, under the null hypothesis that there is cointegration, the residuals $\zeta_{it}$ will also be I(1).

#### 2.2.3. Panel Fully Modified Least Squares Model and Dynamic Ordinary Least Squares Estimator

Subsequently to the cointegration confirmation, the empirical model presented in Equations (1) and (2) could be estimated by applying simple Ordinary Least Squares (OLS), Random Effect, Fixed Effect, or GMM approaches. Nevertheless, these methods can cause discrepancy and ambiguous coefficients when applied to cointegrated panel data [39]. However, the Group Mean Fully Modified Ordinary Least Squares (GM-FMOLS) proposed by Pedroni [40] and the Dynamic Ordinary Least Squares (DOLS) introduced by Stock and Watson [41] are both appropriate methods to avoid this type of inconsistency and misleading of coefficients. Besides, FMOLS is useful to eliminate the problem of regressors’ endogeneity, and serial correlation, which might also result in consistent estimate parameters in a relatively small sample [42]. Similarly, the dilemma of endogeneity, serial correlation, and multicollinearity is solved by using the DOLS method through the inclusion of lags and leads of the differenced I(1) regressors in the regression [41]. Furthermore, the DOLS estimator discloses the cointegrating vector.

#### 2.2.4. Auto Regression Distributed Log Model

The ARDL cointegration method is applied with variables that are integrated of different order, I(0), I(1), or a combination of both, and isrobust in a small sample size when there is a single long run relationship between the underlying variables. The F-statistic (Wald test) is used to detect this long run relationship, which is confirmed when the F-statistic exceeds the critical value band [43].

The general ARDL (*p*,*q_1_*,*q_2_*......*q_k_*) technique is specified by the following equation:

(9) $$\Phi \left(L\right)y_{t}=\varphi +\theta_{1}\left(L\right)x_{1t}+\theta_{2}\left(L\right)x_{2t}+\theta_{k}\left(L\right)x_{kt}+\mu_{t}$$

Using the lag operator *L* applied to each component of a vector, *L^k^y* = *y_t−k_*, it is convenient to define the lag polynomial Φ(*L*,*p*) and the vector polynomial *β*(*L,q*). As long as the error term *u_t_* is a white noise process or, more generally, is stationary and independent of *x_t_*, *x_t_*_−1_, ... and *y_t_*, *y_t_*_−1_, ..., the ARDL models can be estimated consistently by ordinary least squares.

## 3. Results and Discussion

Table 2 displays a summary of the descriptive statistics of the studied variables. It is possible to observe (variables in logs) that they have a similar variability (Std. Dev.) around the Mean, except with REGTAX (registration tax), which presents the highest variability among countries and years, and NEW (new cars registration) depicting the lowest variability around the Mean.

The Panel Unit Root tests stated in Section 2 were applied to all the variables, and the results are presented in Table 3. Most variables are non-stationary in levels, suggesting long-run relationships among them. Nevertheless, all variables are stationary in the first differences.

Table 4 shows the results for Kao and Pedroni cointegration tests, which evidence cointegration across different countries’ panels. The results of both cointegration test values of Kao and Pedroni indicate that cointegration is significant, suggesting that there is cointegration amongst the chosen variables.

Panel Fully Modified Least Squares Model and Dynamic Ordinary Least Squares Estimator results are presented in Table 5. It is possible to observe that in model 1, all the variables have some significance in affecting CO_2_, at least in one of the methods (FMOLS or DOLS). Moreover, GDP and NEW have a positive and significant impact on CO_2_, whereas taxes exert a negative and significant influence on emissions. In model 2, we can observe that GDP exerts a significant and positive impact on NEW, while both taxes have a significant negative impact on it. As in [2], in general, the impact of transport tax is of higher magnitude than the impact of the registration tax because, in their decisions, consumers usually give much more relevance to the immediate purchase price than to annual or monthly duties; that is, transport taxes can be more effective in reducing emissions and the demand for new cars, than the registration tax.

The results reported in Table 6 show the long-run and short-run impacts of the independent variables on CO_2_ emissions and on new cars’ registrations in Mediterranean countries from the ARDL model. The long-run analysis results show that both GDP and REGTAX are positive determinants of CO_2_ at the 1 percent level of significance. Ceteris paribus, a 1 percent increase in GDP and REGTAX raises CO_2_ by 1.52 percent and 0.27 percent, respectively. On the other side, NEW and TRTAX are negative determinants of CO_2_, decreasing it by −0.16 percent and −0.32 percent, respectively, from a 1 percent rise. These results for NEW and REGTAX have different signs from those obtained in FMOLS and DOLS models, which might evidence that in the long run, the new registered cars can be “cleaner”, exerting a negative impact on CO_2_ emissions. The short-run estimations did not reveal any significance in ARDL-simulated models. Once again, TRTAX was revealed to be more effective than REGTAX in decreasing CO_2_ emissions.

Concerning the model 2, in which NEW is the dependent variable, it can be observed that only GDP is significant in the long run, though in the short run, no significance was found in these estimations. Nevertheless, the positive impact of GDP, already found in the previous methodologies, was confirmed, with a raise of 0.6 percent in NEW, resulting from a 1 percent increase in GDP.

## 4. Conclusions

This article intends to assess if observed reductions in CO_2_ emissions in the Mediterranean countries can be attributed to CO_2_-based vehicle taxation. In particular, the impact of car registration taxes and transport taxes paid by households on CO_2_ emissions and on the decision of buying a new car are analyzed for European Mediterranean countries, from 2008 to 2018. We applied econometric techniques as FMOLS, DOLS, and ARDL models.

Our results allow for the conclusion that economic growth encourages the purchase of new cars, which increases CO_2_ emissions. However, in the long run, a negative impact of new cars on emissions is observed, which might reflect a change in the type of vehicles purchased in favor of electric vehicles, or less polluting ones, in the Mediterranean countries. Our findings also give evidence of the relevance of fiscal policies in reducing CO_2_ emissions from transport activities by households, as showed by the significant and negative sign of registration taxes and transport taxes coefficients both on CO_2_ emissions and on the new cars’ registrations. These results are in accordance with [10,22,23,24], although the studies of [22,23] refer to northern European countries. Furthermore, transport taxes were revealed to be more effective in decreasing emissions and the demand for new cars, than the registration tax, as also confirmed by [2].

This study confirms that taxes based on CO_2_ emissions or with a CO_2_ component on passenger vehicles can be useful for policy makers to help reduce greenhouse gas emissions and the consequent impacts on ecosystems and human health. In particular, they can be effective in promoting more efficient and environmentally friendly mobility by encouraging the use of collective transport, and mainly by creating incentives for consumers to purchase less-polluting vehicles. By increasing the production, purchase, and ownership of this type of vehicles, the promotion of electric mobility can be considerably anticipated if governments apply this fiscal policy instrument, especially in the Mediterranean countries.

The transport sector is still responsible for a large share of CO_2_ emissions in Europe. Considering that our results show that in the long run, consumers can change their consumption patterns from more polluting to, potentially, electrical vehicles, these kind of instruments will remain crucial in the future for the Mediterranean countries to meet the EU targets on emissions and comply with the objectives of the Paris Agreement.

For their better accomplishment, these instruments should be complemented by other types of coordinated measures and actions, not only at the national level, but also at the international level. The contribution of this work to the existing literature in this area helps clarify the path that different governments must follow and supports policy makers achieving the necessary harmonization of this tax policy within the EU.

## Figures and Tables

**Table 1:** Vehicle Taxes in the Mediterranean Countries.

Country	Registration Tax	Annual Circulation Tax
Bulgaria		Centered on the EU emission standard (not directly linked to CO_2_ emissions)
Croatia	CO_2_-based emissions, purchase price, and fuel type	
Cyprus	CO_2_-based taxation	CO_2_-based taxation
France	Bonus/malus system centered on CO_2_ emissions: Bonus: cars or vans equal or under 20 g CO_2_/km emissions (max 6000€). Malus: starting at 50€ (for 138 g CO_2_/km) until a maximum of 20,000€ (>213 g CO_2_/km). Scrapping scheme based on CO_2_ to replace old vehicles by low-emission new ones (below 116 g CO_2_/km)	Annual malus: 160€ for vehicles emitting above 190 g CO_2_/km
Greece	CO_2_-based: coefficient ranges between 0.95 (under 100 g CO_2_/km) and 2.00 (>250 g CO_2_/km)	CO_2_-based (vehicles registered after 31 October 2010): Values range between 0.90€/g of CO_2_ released (91–100 g CO_2_/km) and 3.72€/g (>251 g CO_2_/km) Exempt to vehicles under 90 g CO_2_/km
Italy	Bonus/malus system based on CO_2_ emissions: Bonus: one-off amount (max 6000€) vehicles under 20 g CO_2_/km at first registration, between March 2019 and the end of December 2021 Malus: up to max 2500€ (>250 g CO_2_/km)	
Malta	CO_2_-based taxation: ((X% + CO_2_ * RV) + (Y% + length + RV)) x% = based on CO_2_ Y% = based on the length of the car REV = vehicle registration value	Based both on the CO_2_ emissions and the age of the car In the first 5 years, taxation depends on the CO_2_ emissions only, ranging between 100€ (for emissions up to 100 g CO_2_/km) and 180€ (for emissions 150–180 g CO_2_/km)
Portugal	Environmental tax component based on CO_2_: Lowest rate: under 110 g CO_2_/km gasoline vehicles pay ((0.40 * CO_2_) − 39); diesel vehicles pay ((1.56 * CO_2_) − 10.43) Highest rate: gasoline vehicles over 235 g CO_2_/km pay ((212 * CO_2_) − 38,000); diesel vehicles over 190 g CO_2_/km pay ((256 * CO_2_) − 34,700)	Environmental tax component based on CO_2_ for vehicles until 2.5 tonnes registered after 1 July 2007
Romania	Scrapping scheme based on CO_2_: incentive to replace vehicles older than 8 years by low-emission (under 96 g CO_2_/km) or zero-emission vehicles	
Slovenia	CO_2_-based taxation: ranges from 0.5% (gasoline) and 1% (diesel) under 110 g CO_2_/km to 28% (gasoline) and 31% (diesel) over 250 g CO_2_/km Incentives based on CO_2_ for electric vehicles	
Spain	CO_2_-based taxation: ranges between 5.4% (120-160 g CO_2_/km) and 16.9% (200 g CO_2_/km and more)	Based on fuel efficiency (not directly associated to CO_2_ emissions): 75% tax-reduction for fuel-efficient vehicles in the most important cities (e.g., Madrid, Barcelona, Valencia)

Source: ACEA (2020).

**Table 2:** Descriptive statistics findings.

	**CO_2_**	**GDP**	**NEW**	**REGTAX**	**TRTAX**
Mean	19,873,624	40,4063.8	526,124	476.41	1402.56
Median	6,660,100	109,269.3	153,847	136.5	294.88
Maximum	71,252,970	1,941,829	2,269,011	2326	8204.29
Minimum	164,550	5748.11	9542	0.0	11.05
Std. Dev.	25,795,646	590,279.6	718,432.7	672.16	2201.8
Observations	121	121	121	121	121
	**LCO_2_**	**LGDP**	**LNEW**	**LREGTAX**	**LTRTAX**
Mean	15.64	11.61	12.08	4.73	6.01
Median	15.71	11.60	11.94	5.02	5.69
Maximum	18.08	14.48	14.63	7.75	9.01
Minimum	12.01	8.66	9.16	−2.30	2.40
Std. Dev.	1.76	1.72	1.58	2.12	1.67
Observations	121	121	121	121	121

**Table 3:** Panel Unit Root Tests Results.

	Levels				First Differences			
	LLC	IPS	ADF	PP	LLC	IPS	ADF	PP
CO_2_	−2.17 **	0.16	54.12	85.14	−5.55 ***	−2.22 **	76.06 **	127.79 ***
GDP	3.41	4.99	17.1	38.25	−3.16 ***	−2.26 **	82.18 ***	236.73 ***
NEW	0.26	0.06	57.01	81.87 ***	−0.14 ***	−2.70 ***	81.78 ***	244.58 ***
REGTAX	−1.33 *	0.50	43.77	118.96 ***	−3.08 ***	−2.17 **	72.73 ***	204.13 ***
TRTAX	−1.89 **	0.39	46.88	119.64 ***	−6.33 ***	−3.40 ***	53.79 ***	98.78 ***

Note: *** *p* < 0.01; ** *p* < 0.05; * *p* < 0.1.

**Table 4:** Panel Cointegration Tests Results.

	Statistic	*p* Value
Kao cointegration test	−3.90	0.0000
Augmented Dickey Fuller t	−3.55	0.0002
Pedroni cointegration test	−3.73	0.0001
Phillips-Perron t	−4.98	0.0000

**Table 5:** Panel Fully Modified Least Squares Model and Dynamic Ordinary Least Squares Estimator Results.

LCO_2_ Dependent (Model 1)			LNEW Dependent (Model 2)		
Variables	FMOLS	DOLS	Variables	FMOLS	DOLS
LGDP	1.74 *** (0.00)	−0.08 (0.77)	LGDP	1.27 *** (0.00)	1.07 *** (0.00)
LNEW	−0.16 (0.39)	1.70 *** (0.00)			
LREGTAX	−0.26 *** (0.00)	−0.28 *** (0.01)	LREGTAX	−0.12 * (0.06)	−0.07 (0.26)
LTRTAX	−0.24 * (0.09)	−0.31 ** (0.05)	LTRTAX	−0.17 * (0.08)	−0.12 (0.23)

Note: *p* values are reported in parentheses; *, **, and *** represent the significance level of 10%, 5%, and 1%, respectively.

**Table 6:** Auto Regression Distributed Log Model results.

LCO_2_ Dependent (Model 1)			LNEW Dependent (Model 2)		
Variables	Long Run	Short Run	Variables	Long Run	Short Run
COINTEQ01		−0.18 * (0.02)	COINTEQ01		−0.14 * (0.069)
LNEW	−0.16 *** (0.00)	0.06 (0.34)			
LGDP	1.52 *** (0.00)	−0.24 (0.34)	LGDP	1.17 *** (0.00)	0.02 (0.98)
LREGTAX	0.27 *** (0.00)	0.01 (0.86)	LREGTAX	−0.69 *** (0.00)	0.22 (0.29)
LTRTAX	−0.32 *** (0.00)	0.21 (0.14)	LTRTAX	0.28 ** (0.02)	0.23 (0.72)

Note: *p* values are reported in parentheses; *, **. and *** represent the significance level of 10%, 5%, and 1%, respectively.

## Data Availability

Publicly available datasets were analyzed in this study. These data can be found here: https://ec.europa.eu/eurostat/web/main (accessed on 30 September 2020).
